# Correction: New multifunctional hybrids as modulators of apoptosis markers and topoisomerase II in breast cancer therapy: synthesis, characterization, and *in vitro* and *in silico* studies

**DOI:** 10.1039/d4ra90101k

**Published:** 2024-09-19

**Authors:** Heba W. Alhamdi, Mohammad Y. Alfaifi, Ali A. Shati, Serag Eldin I. Elbehairi, Mohammed Er-rajy, Reda F. M. Elshaarawy, Yasser A. Hassan, Rozan Zakrya

**Affiliations:** a College of Sciences, Biology Department, King Khalid University Abha 61413 Saudi Arabia; b King Khalid University, Faculty of Science, Biology Department Abha 9004 Saudi Arabia; c Tissue Culture and Cancer Biology Research Laboratory, King Khalid University Abha 9004 Saudi Arabia; d Cell Culture Lab, Egyptian Organization for Biological Products and Vaccines (VACSERA Holding Company) 51 Wezaret El-Zeraa St. Agouza Giza Egypt; e LIMAS Laboratory, Faculty of Sciences Dhar El Mahraz, Sidi Mohamed Ben Abdellah University Fez Morocco; f Department of Chemistry, Faculty of Science, Suez University 43533 Suez Egypt reda.elshaarawy@suezuniv.edu.eg; g College of Pharmacy, Al-Kitab University Kirkuk 36015 Iraq; h Department of Pharmaceutics, Faculty of Pharmacy, Delta University for Science and Technology Gamasa Egypt; i Chemistry Department, Faculty of Science, Port-Said University Port-Said Egypt rzakrya@yahoo.com

## Abstract

Correction for ‘New multifunctional hybrids as modulators of apoptosis markers and topoisomerase II in breast cancer therapy: synthesis, characterization, and *in vitro* and *in silico* studies’ by Heba W. Alhamdi *et al.*, *RSC Adv.*, 2024, **14**, 28555–28568, https://doi.org/10.1039/D4RA04219K.

The authors regret the omission of one of the affiliations (affiliation *g*) from the original manuscript. The corrected list of affiliations and author attributions is as shown above.

The Royal Society of Chemistry apologises for these errors and any consequent inconvenience to authors and readers.

